# Trimester-specific reference ranges for thyroid hormones in pregnant women

**DOI:** 10.1097/MD.0000000000014245

**Published:** 2019-01-25

**Authors:** Daowen Zhang, Keying Cai, Guixia Wang, Shuhang Xu, Xiaodong Mao, Ang Zheng, Chao Liu, Kuanlu Fan

**Affiliations:** a Department of Endocrinology, The Second Affiliated Hospital of Xuzhou Medical University, Xuzhou; b Department of Endocrinology and Metabolism, Affiliated Hospital of Integrated Traditional Chinese and Western Medicine, Nanjing University of Chinese Medicine, Nanjing, China.

**Keywords:** pregnancy, reference range, thyroid disease, thyroid function

## Abstract

The aim of this study was to determine the trimester-specific reference range of thyroid function in Nanjing.

A total of 805 pregnant women in the 1st, 2nd, and 3rd trimesters were recruited in the prospective, observational study during their routine antenatal clinic visit and 282 nonpregnant subjects served as controls. A questionnaire was completed by the subjects to record their personal health history, family history of thyroid disease, and consumption of estrogen or antithyroid drugs. Thyroid palpation was performed to exclude the thyroid goiter. Thyroid function and urine iodine were measured by chemiluminescence and arsenic cerium analysis.

The trimester-specific reference ranges in Nanjing were as follows: thyroid-stimulating hormone (TSH) 0.02 to 3.78 mIU/L, free thyroxine (FT4) 13.93 to 26.49 pmol/L, total thyroxine (TT4) 103.39 to 319.43 nmol/L in the 1st trimester. TSH 0.47 to 3.89 mIU/L, FT4 12.33 to 19.33 pmol/L, TT4 92.28 to 234.88 nmol/L in the 2nd trimester. TSH 0.55 to 4.91 mIU/L, FT4 11.38 to 19.21 pmol/L, TT4 83.54 to 258.12 nmol/L in the 3rd trimester. According to the TSH reference range recommended by American Thyroid Association (ATA), the prevalence of subclinical hypothyroidism, subclinical hyperthyroidism, hyperthyroidism, hypothyroxinemia, and thyroid peroxidase antibody-positive were 12.42%, 0.50%, 0.99%, 1.61%, and 11.80%, respectively, prevalence according to the trimester-specific reference range were 1.99%, 0.25%, 1.61%, 0.37%, and 1.61%, respectively, which showed elevated hypothyroxinemia incidence and declined incidence of subclinical hypothyroidism and hyperthyroidism.

Trimester-specific reference range varied from that of ATA's recommendation, influencing the diagnosis, and treatment of pregnant thyroid disorders. To detect and control these disorders properly, setting up trimester-specific reference is clinically essential.

## 1 Introduction

Thyroid hormone plays a major role in the growth and development of humans, such as nervous system development and skeletal muscle growth. Thyroid hormone for fetal growth and development are mainly dependent on the stage of pregnancy, especially in the 1st trimester. Maternal thyroid hormones deficiency may cause severe obstetric outcomes, such as fetal developmental abnormalities, nerve function abnormalities, spontaneous abortions, and stillbirth.^[1–3]^ Due to a variety of factors, such as iodine nutrition, race, human chorionic gonadotropin (HCG) secretion, and thyroid autoantibodies, pregnancy thyroid hormone is continuously changing. According to the “2017 Guidelines of the American Thyroid Association for the Diagnosis and Management of Thyroid Disease During Pregnancy and the Postpartum” (termed as Guideline henceforth) published by American Thyroid Association (ATA) in 2017,^[4]^ a reduction in the lower thyroid-stimulating hormone (TSH) reference range is observed during pregnancy, there is significant geographic and ethnic diversity in TSH concentrations during pregnancy, “population-based trimester-specific reference ranges for serum TSH should be defined through assessment of local population data representative of a healthcare provider's practice, reference range determinations should only include pregnant women with no known thyroid disease, optimal iodine intake, and negative TPOAb status” is recommended. Before the trimester-specific thyroid normal reference range in each regions, the TSH upper reference limit of 2.5 mIU/L in the 1st trimester, and 3.0 mIU/L in the 2nd and 3rd trimesters was adopted, which is not properly. To reasonably detect and control the thyroid dysfunctions during pregnancy, a trimester-specific thyroid normal reference range is required in different regions and laboratories. The present study recruited a total of 805 pregnant women during their routine prenatal visit. We established a trimester-specific reference range of thyroid function for pregnant women, and then compared prevalence of thyroid dysfunction based on the new trimester-specific reference range and ATA standard.

## 2 Subjects and methods

### 2.1 Study population

In this prospective, observational study, we recruited 805 pregnant women of childbearing age during their routine visits between July 11 and November 28, 2012. The participants in the 1st trimester (1–12 weeks of pregnancy) research were recruited from Nanjing Nanhu Community Service Center, Xinglong Community Service Center, Nanyuan Community Service Center and Binhu Community Service Center; participants in the 2nd and 3rd trimester studies were from the Obstetrics and Gynecology Clinic of Affiliated Hospital of Integrated Traditional Chinese and Western Medicine (13–27 and 28–40 weeks, respectively). The Medical Ethics Committee of the Affiliated Hospital of Integrated Traditional Chinese and Western Medicine approved the study, and informed consent was obtained from all subjects. Participants in the nonpregnant control group consisted of nonpregnant women attending regular check-up at Affiliated Hospital of Integrated Traditional Chinese and Western Medicine. Finally, 1087 women were recruited in the study. Of these, 282 comprised the nonpregnant control group, and 288, 255, and 262 were in the 1st, 2nd, and 3rd trimester groups for pregnant participants group.

High iodine food intake was not permitted 2 weeks before the visit, such as kelp, laver, and jellyfish, etc. Also, drinking excess water was not allowed 2 hours before the urine collection. Exclusion criteria: personal or family history of thyroid diseases; visible or palpable goiter; abnormal liver, kidney, or heart function; usage of estrogen or antithyroid drugs.

### 2.2 Methods

#### 2.2.1 Questionnaire

A questionnaire in Chinese was drafted to investigate the general information of the participants. The details of demographic, clinical, and family history of participants were recorded, especially personal and family history of thyroid diseases, or positive thyroid autoimmune antibodies, visible or palpable goiter, abnormal function of liver, kidney or heart, and personal history of mediation use.

#### 2.2.2 Sample collection

At least 10 mL midstream urine was collected, of which 1.5 mL was stored at 4°C. Three milliliter of blood samples were obtained from each participant on the morning after overnight fast, and the isolated serum samples were stored at 4°C. Thyroid function [TSH, triiodothyronine (TT3), thyroxine (TT4), free triiodothyronine (FT3), free thyroxine (FT4), thyroid peroxidase antibody (TPOAb)] was measured within 24 hours.

#### 2.2.3 Measurement of thyroid function

The TSH, FT3, FT4, TT3, TT4, and TPOAb were measured by chemiluminescence. Instruments and reagents were provided by Siemens Company (Centaur CP, Germany). The functional sensitivity of the TSH assay was 0.02 mIU/L. The intra-assay coefficient of variations (CVs) of serum TSH, FT4, FT3, and TPOAb were 1.57% to 4.12%, 2.24% to 6.33%, 0.57% to 4.31%, and 2.42% to 5.63%, respectively. The inter-assay CV values were 1.26% to 5.76%, 4.53% to 8.23%, 3.50% to 5.19%, and 5.23% to 8.16% respectively. The urinary iodine excretion was measured by the colorimetric ceric ion arsenious acid ash method, based on the Sandell–Kolthoff reaction. The intra- and inter-assay CV values were 7.0%.

### 2.3 Diagnostic criteria

#### 2.3.1 Iodine nutrition

The iodine reference range for pregnancy is slightly different from that for adults. The urine iodine concentration (UIC) < 150 μg/L, 150 μg/L ≤ UIC < 250 μg/L, 250 μg/L ≤ UIC < 500 μg/L, and UIC ≥ 500 μg/L are defined as iodine deficiency, adequate, more than adequate, and excess, respectively. Iodine adequate is considered as optimal iodine nutrition according to World Health Organization (WHO) recommendations and 2017 Guidelines of the American Thyroid Association for the Diagnosis and Management of Thyroid Disease During Pregnancy and the Postpartum.^[4]^

#### 2.3.2 Reference range for thyroid function

The reference range of Affiliated Hospital of Integrated of Traditional Chinese and Western Medicine were offered by the clinical laboratory as follows: TSH 0.55 to 4.78 μIU/L, FT3 3.5 to 6.5 pmol/L, FT4 11.5 to 22.7 pmol/L, TT3 0.92 to 2.79 nmol/L, TT4 24.5 to 171.6 nmol/L, TPOAb 0 to 60 IU/mL.

This study was performed in 2012, so the TSH reference range was based on Guideline of ATA established in 2012. “Guidelines of the American Thyroid Association for the Diagnosis and Management of Thyroid Disease During Pregnancy and Postpartum,” TSH upper reference limit of 2.5 mIU/L in the 1st trimester, and 3.0 mIU/L in the 2nd and 3rd trimesters.

To establish a trimester-specific normal reference range of thyroid function for pregnant women, a minimum of 120 pregnant women are needed. Inclusion criteria: age ≥18 but ≤40 years; no personal history of thyroid diseases; no visible or palpable goiter; no liver/kidney/heart dysfunction; no family history of thyroid diseases; no medication history of estrogen or antithyroid drugs; and optimal iodine nutrition.^[5]^ Exclusion criteria: Thyroid function test, urine iodine, or questionnaire was incomplete. The 2.5% to 97.5% confidence interval of each thyroid function index in the 3 trimesters would be trimester-specific thyroid normal reference range, respectively. Isolated hypothyroxinemia is defined as a normal maternal TSH concentration in conjunction with FT4 levels in the lower 5th or 10th percentile of the reference range.

The study had already been signed up on the website http://www.medresman.org/ on March 3, 2014; Registration number is ChiCTR-ECS-14004317.

### 2.4 Statistical analysis

All statistical analyses were performed using the Statistical Package for Social Sciences and Problem Solutions (SPSS, version 16.0). *P* < .05 indicated statistically significant difference. Chi-squared test was used to compare the 2 different rates. Mean (*X*) represented the central tendency of quantitative data, and standard deviation (*S*) represented the discrete tendency.

## 3 Results

### 3.1 Baseline characteristics

A total of 1087 women were recruited in the study. Of these, 282 were enrolled in the nonpregnant control group, with an average age 26.88 ± 3.10 years; 288 in the 1st trimester group, 27.73 ± 3.41 years; 255 in 2nd trimester group, 26.60 ± 3.67 years; and 262 in the 3rd trimester group, 26.33 ± 3.77 years (Table 1). The median UICs of the 1st, 2nd, and 3rd trimester groups were 180.7, 170.1, and 165.4 μg/L, respectively, indicating the iodine nutrition of the pregnant women in Nanjing is adequate.

**Table 1:**
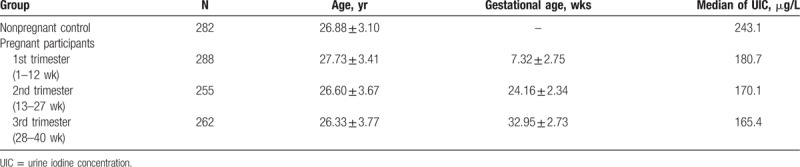
Baseline of each groups.

### 3.2 Trimester-specific reference range of thyroid function

To establish a trimester-specific reference range of thyroid function, 132, 124, and 127 eligible pregnant women in 3 trimesters after screened in each trimester group, respectively, meanwhile 125 eligible women in nonpregnant control group. The trimester-specific reference range of thyroid function in Nanjing is listed in Table 2. The TSH reference range in nonpregnant control group, 1st trimester, 2nd trimester, and 3rd trimester is 0.76 to 4.88, 0.02 to 3.78, 0.47 to 3.89, and 0.55 to 4.91 mIU/L, respectively; the nonpregnant control group is close to the laboratory criteria. The TSH reference range in 1st trimester was above 2.5 mIU/L, 2nd and 3rd trimester criteria were above 3.0 mIU/L, latter is recommended by ATA. The tendency of TSH index increased along with the trimester, and approached the nonpregnant control group criteria in the 3rd trimester.

**Table 2:**
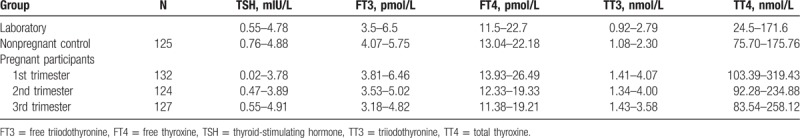
Established of trimester-specific reference range of thyroid function in each groups.

**Figure 1:**
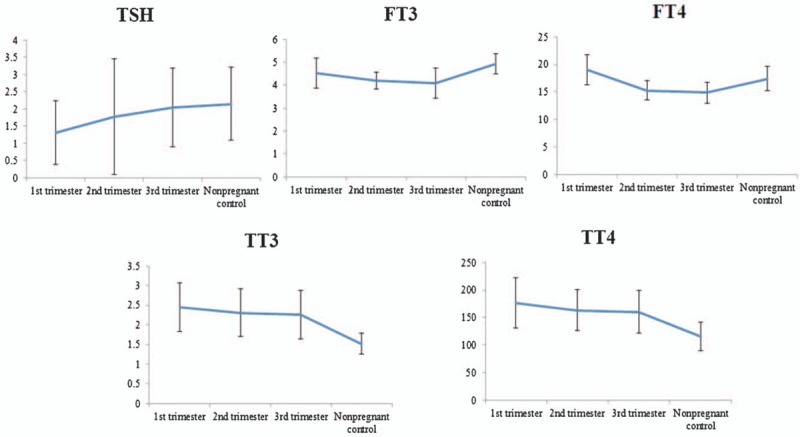
Trend of thyroid hormones level in each trimester during of pregnancy. FT3 = free triiodothyronine, FT4 = free thyroxine, TPOAb = thyroid peroxidase antibody, TT3 = triiodothyronine, TT4 = thyroxine, TSH = thyroid-stimulating hormone.

In addition, The FT4 reference range in nonpregnant control group, 1st trimester, 2nd trimester, and 3rd trimester is 13.04 to 22.18, 13.93 to 26.49, 12.33 to 19.33, and 11.38 to 19.21 pmol/L, respectively, the upper limit and lower limit both declining along with the trimester, higher than nonpregnant control group in 1st trimester, afterwards lower than nonpregnant control group in 3rd trimester. The TT4 reference range in nonpregnant control group, 1st trimester, 2nd trimester, and 3rd trimester is 75.70 to 175.76, 103.39 to 319.43, 92.28 to 234.88, and 83.54 to 258.12 nmol/L, the upper limit and lower limit also declining along with the trimester, but always higher than nonpregnant control group (Fig. 1). Our study indicated that TT4 mean value of the 1st trimester was 1.82-fold than the nonpregnant control group, declining to 1.39 to 1.51-fold in 2nd and 3rd trimesters.

### 3.3 Prevalence of thyroid dysfunctions in each trimester based on trimester-specific reference range of thyroid function

The frequencies of each thyroid dysfunction are summarized in Table 3. Among 805 pregnant women, 70 (8.70%) were found to have thyroid dysfunction, meanwhile, 20 (7.09%) women in 282 of nonpregnant control group. The prevalence of thyroid dysfunction between pregnant women and control was not significantly different (*P* = .603). Based on the trimester-specific reference range of thyroid function, 16 (1.99%) women were diagnosed as subclinical hypothyroidism; of whom, 3, 10, and 3 were in the 1st, 2nd, and 3rd trimesters, respectively. Only 1 (6.25%) was TPOAb positive. In addition, 2 (0.25%) exhibited overt hypothyroidism. Each of whom, 1, 1 were in the 1st and 2nd trimesters, and 1 (50.00%) was TPOAb positive.

**Table 3:**
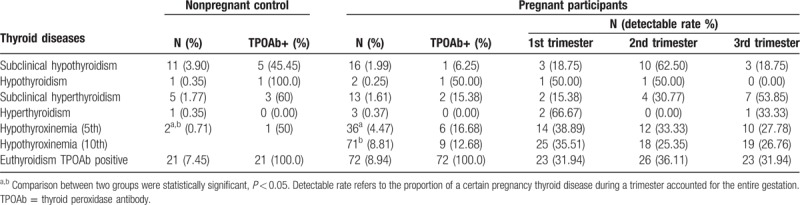
Prevalence of thyroid diseases in each trimester by trimester-specific reference range.

Thirteen (1.61%) women had subclinical hyperthyroidism; 2, 4, and 7 pregnant women were in the 1st, 2nd, and 3rd trimesters, respectively. Two (15.38%) were TPOAb positive. There were 3 (0.37%) overt hyperthyroidism pregnant women; 2 and 1 pregnant women were in the 1st and 3rd trimesters, respectively. None were TPOAb positive. Seventy-one (8.81%) had hypothyroxinemia (10th); 25, 18, and 19 pregnant women were in the 1st, 2nd, and 3rd trimesters, respectively, and 9 (12.68%) were TPOAb positive. Fewer women (36, 4.47%) had hypothyroxinemia (5th); 14, 12, and 10 pregnant women were in the 1st, 2nd, and 3rd trimesters, respectively, and 6 (16.68%) were TPOAb positive. A total of 95 (13.55%), pregnant women were TPOAb positive; of which, 72 (8.94%) had euthyroidism, 23, 23, and 23 pregnant women were in the 1st, 2nd, and 3rd trimesters, respectively.

### 3.4 Prevalence of thyroid dysfunctions in each trimester based on ATA criteria

The frequencies of each thyroid dysfunction are summarized in Table 4. Among 805 pregnant women, 133 (16.52%) were found to have thyroid dysfunction, meanwhile, 20 (7.09%) women in 282 of nonpregnant control group. The prevalence of thyroid dysfunction between pregnant women and control was significantly different (*P* = .001). Based on ATA criteria, 100 (12.42%) were diagnosed as subclinical hypothyroidism; of whom, 39, 27, and 34 were in the 1st, 2nd, and 3rd trimesters, respectively, 18 (18.00%) were TPOAb-positive. In addition, 4 (0.50%) had overt hypothyroidism, each of whom, 1, 1, and 2 were in the 1st, 2nd, and 3rd trimesters, respectively, and only 1 (25.00%) was TPOAb positive. Eight (0.99%) pregnant women had subclinical hyperthyroidism, each of whom, 7 and 1 were in 1st and 2nd trimesters, respectively, only 1 (12.50%) was TPOAb positive.

**Table 4:**
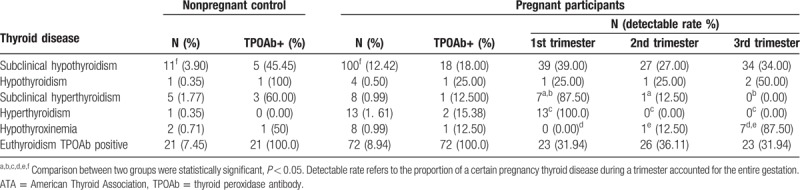
Prevalence of thyroid diseases in women by ATA reference range.

Thirteen (1.61%) women had overt hyperthyroidism pregnant women; 13 in the 1st trimester, and 2 (15.38%) were TPOAb positive. Eight (0.99%) had hypothyroxinemia; of these, 1 and 7 pregnant women were in the 2nd and 3rd trimesters, respectively, and 1 (12.50%) pregnant woman was TPOAb positive. A total of 95 (13.55%) pregnant women were TPOAb positive, of which, 72 (8.94%) had euthyroidism, 23, 23, and 23 pregnant women were in the 1st, 2nd, and 3rd trimesters, respectively.

Comparing the thyroid dysfunctions from each trimester using Chi-squared test, we found that the prevalence of subclinical hypothyroidism in the TPOAb-positive group was higher than the TPOAb-negative group (*P* = .001). Besides, the prevalence of subclinical hyperthyroidism and overt hyperthyroidism in the 1st trimester was higher than those in the 2nd and 3rd (*P* = .008, *P* = .008, *P* = .033, *P* = .008), and the prevalence of hypothyroxinemia in the 3rd trimester was greater than that in the 1st and 2nd (*P* = .015, *P* = .002) (Table 4).

There were 282 women recruited as control. Among them, 11 (3.90%) presented subclinical hypothyroidism; and 5 (45.45%) were TPOAb positive. One (0.35%) had overt hypothyroidism with positive TPOAb (100.00%). And 5 (1.77%) women exhibited subclinical hyperthyroidism, 3 (60.00%) of them were TPOAb positive. There was 1 (0.35%) overt hyperthyroidism pregnant woman without TPOAb positive (0.00%). Two (0.71%) women demonstrated hypothyroxinemia, 1 (50.00%) of them was TPOAb positive. A total of 41 (14.54%) women were TPOAb positive. Compared to the pregnant participants group, using Chi-squared test, the nonpregnant control group had a significantly lower prevalence of subclinical hypothyroidism (*P* = .001). The nonpregnant control group had a higher prevalence of TPOAb but did not achieve statistical significance (*P* = .232) (Table 4).

### 3.5 Comparison between 2 reference ranges

Compared to the diagnostic criteria recommend by ATA, the prevalence of subclinical hypothyroidism was significantly lower by the trimester-specific reference range (1.99% and 12.42%, respectively); however, it was higher in hypothyroxinemia [4.47% (5th) or 8.81% (10th) vs 0.99%, *P* = .001, *P* = .001]. There were no significant differences of the prevalence of hypothyroidism (0.25% vs 0.50%, *P* = .278), subclinical hyperthyroidism (0.37% vs 1.61%, *P* = .278), hyperthyroidism (0.37% vs 1.61%, *P* = .013) between these 2 reference ranges of thyroid function.

## 4 Discussion

The thyroid function during pregnancy has been shown to be influenced by HCG secretion, fetal thyroid hormone use, and altered during gestation. Among these, TSH and TT4 are the most significant. Recommended by ATA, the upper limit of TSH is 2.5, 3.0, and 3.0 mIU/L in the 3 trimesters, respectively. Thyroid function during pregnancy varies with race, iodine nutrition, and test method. Therefore, an establishment of trimester-specific thyroid normal reference ranges in different regions and laboratories are highly recommended. The present study recruited pregnant women from Nanjing with no personal and family history of thyroid diseases, or positive thyroid autoimmune antibodies, visible or palpable goiter, optimal iodine nutrition, abnormal function of liver, kidney or heart, and personal history of mediation use.

Since 125 women in the nonpregnant control group, 132 in the 1st trimester, 124 in the 2nd trimester, and 127 in the 3rd trimester were enrolled, it fulfilled the quantity standards to establish a reference range. During pregnancy, HCG secreted by placenta trophocyte commences on the 7th day of gestation, peak in 8–10^th^ week, and then declining after lasting 1–2 weeks, until the end. HCG demonstrated a similar role to TSH, and thus, TSH 1st decreased and then increased, correspondingly. In our study, the upper limit, lower limit, and mean values of TSH in the 3 trimesters ascended gradually; the upper limit of the 3rd trimester approached the nonpregnant control group, which were in agreement with the previous studies.^[6–9]^ In addition, estrogen exerted the effects of thyroid-binding globulin synthesis, which increases TT4 during gestation by 1.5- to 2-fold, approximately 50% for the fetus. Because T4 are transported through the placenta into the fetus, the maternal T4 level gradually declines, resulting into a possible hypothyroxinemia. Our study demonstrated that TT4 value of the 1st trimester was 1.82-fold than the nonpregnant control group, declining to 1.39- to 1.51-fold in 2nd and 3rd trimesters, consistent with the previous studies.^[10]^ We also established the normal reference range for the nonpregnant control group, which is similar as that established in our laboratory. The reference range of TSH in our study was higher than that of ATA, correlated to the race, region, and inspection method.

Secondly, according to the pregnancy reference range recommended by ATA, the prevalence of subclinical hypothyroidism, hypothyroidism, subclinical hyperthyroidism, hyperthyroidism, hypothyroxinemia, and TPOAb positive was 12.42%, 0.50%, 0.99%, 1.61%, 0.99%, and 11.80%, respectively. However, based on the trimester reference range of our study, these thyroid disorders were 1.99%, 0.25%, 1.61%, 0.37%, and 8.81%. The prevalence of subclinical hypothyroidism and hyperthyroidism was lower than that of the ATA standard, and hypothyroxinemia was higher. Also, the upper limit of TSH during 3 trimesters was elevated, the upper limit of FT4 in 1st and 2nd trimesters was elevated, FT4 lower limit was reduced in the 3rd trimester. Taken altogether, these form a part of the diagnosed thyroid disorders which were based on FT4 and TSH altered values.

The prevalence of subclinical hypothyroidism based on trimester reference range of our study was lower than the ATA standard, which indicates that part of euthyroidism during pregnancy was diagnosed with subclinical hypothyroidism, and even treated with thyroid hormone replacement. Hyperthyroidism may exert an adverse influence on infants.^[11]^ Indeed, some euthyroidism pregnancies based on trimester reference range of our study were diagnosed with hyperthyroidism based on ATA standard, antithyroid drag was not given fortunately. Mild hyperthyroidism in pregnancy does not need to be treated. In addition, a declining FT4 lower limit leads to the risk of over diagnosis of hypothyroxinemia and overtreatment with thyroid hormone replacement. Although an early intervention for hypothyroxinemia is still not recommended in the latest guideline and study,^[4,12]^ further study found that hypothyroxinemia might also adversely affect the infant intelligence development^[13]^; however, an in-depth investigation is essential. Therefore, to avoid adverse pregnancy outcomes, abnormal fetal development, and to screen thyroid dysfunction in an early stage, it is necessary for each laboratory from different regions to establish a trimester-specific reference range for thyroid function and apply it in clinical practice.

Lastly, there are some limitations in our studies. The sample size for each group was relatively small. Participants in 3 trimesters were recruited from different centers. Fortunately, thyroid function was measured in the same laboratory and the comparison among them is plausible and imperative after establishing a trimester reference range.

## 5 Conclusion

Thyroid function during pregnancy plays a vital role in pregnancy outcomes and fetal growth. Thus, accurate diagnosis is particularly important. However, influenced by race, iodine nutrition and test methods, the trimester-specific reference range of thyroid function might very different. It influences the correct diagnosis, thus causing delay or incorrect treatment. The 2017 Guidelines of the American Thyroid Association for the Diagnosis and Management of Thyroid Disease During Pregnancy and the Postpartum recommended that in order to make the detection and controlling of pregnancy thyroid disorder more reasonable, trimester-specific thyroid function reference ranges were essential in different regions and laboratories. The established trimester-specific reference ranges of thyroid function in Nanjing of our present study may be very useful in the management of pregnancy thyroid abnormalities for pregnant women.

## Author contributions

**Data curation:** Daowen Zhang.

**Formal analysis:** Daowen Zhang.

**Investigation:** Daowen Zhang, Guixia Wang, Xiaodong Mao, Ang Zheng.

**Methodology:** Daowen Zhang.

**Software:** Daowen Zhang, Ang Zheng.

**Writing – original draft:** Daowen Zhang.

**Supervision:** Keying Cai, Chao Liu, Kuanlu Fan.

**Visualization:** Keying Cai.

**Writing – review & editing:** Shuhang Xu.

**Resources:** Xiaodong Mao.

**Conceptualization:** Chao Liu.

**Funding acquisition:** Chao Liu.

Daowen Zhang orcid: 0000-0002-1388-5130.
